# HIV/AIDS knowledge and uptake of HIV counselling and testing among undergraduate private university students in Accra, Ghana

**DOI:** 10.1186/1742-4755-10-17

**Published:** 2013-03-28

**Authors:** Kwaku Oppong Asante

**Affiliations:** 1 Department of Human Development and Psychology, Regent University College of Science and Technology, P. O. Box DS 1636, Dansoman, Accra, Ghana

**Keywords:** HIV/AIDS, Counselling and testing, University students, Ghana

## Abstract

**Background:**

HIV Counselling and Testing (VCT) and knowledge about HIV are some key strategies in the prevention and control of HIV/AIDS in Ghana. However, HIV knowledge and utilization of VCT services among university students is low. The main objective was to determine the level of HIV/AIDS knowledge and to explore factors associated with the use HIV counselling and testing among private university students in Accra, Ghana.

**Materials and methods:**

A cross-sectional study was conducted using structured questionnaires among 324 conveniently selected students enrolled at a privately owned tertiary institution in Accra, Ghana.

**Results:**

The respondents consisted of 56.2% males and 43.8% females aged 17 – 37 years. The mean HIV/AIDS knowledge score of was 7.70. There was a significant difference in knowledge of HIV/AIDS by gender where female students had more knowledge about HIV/AIDS than males [t (322) = 2.40, p = 0.017]. The ANOVA results showed that there was a significant difference in HIV/AIDS knowledge according to the age groups [F (3, 321) = 6.26, p = 0. 0001] and marital status [F (3, 321) = 4.86, p = 0. 008] of the sample. Over half of the participants had not tested for HIV, although over 95% of them knew where to access counseling and testing services. The study also revealed a significant association between demographic variables, testing for HIV and intention to test in the future. Participants who were never married (single), aged 17 – 20 years and had knowledge of two routes of HIV transmission were more likely to have taken an HIV test. Males were more likely to take an HIV test in the future than females. Majority of the students receive HIV/AIDS information from both print and electronic media, but few of them received such information from parents.

**Conclusion:**

The students HIV knowledge was very good, yet HIV testing were low. Health education and HIV intervention programmes must not only provide accurate information, but must be made to help to equip private university students, especially females to test for HIV consistently.

## Background

The spread of HIV/AIDS among the productive age group especially among young adults is a public health concern in Ghana. A more serious challenge today, is the growing infection rates among the adolescents in sub-Saharan Africa, which has just over 10% of the world's population, remains the most seriously affected region [1]. Research has shown that the highest group found to be infected with the virus is the age-group 15 to 24 [2]. This adolescent high-risk group accounts for 60 percent of all new infections in many countries [3]. Research in Ghana indicates that the lifestyles of students on university campuses are placing them at risk of contracting the HIV [4,5] as the university environment has been shown to promote sexual activity among the general student population [6,7]. It is therefore not surprising that sexual intercourse has become the most predominant mode of transmission of HIV in sub-Saharan Africa, accounting for approximately 90% of all infections [1].

Additionally, the pressure from fellow students to live up to the standard such as buying latest mobile phones, expensive clothes, jewelry has been shown to influence young women to engage in transactional sex [8,9]. Further research has indicated that although knowledge about a disease is a prerequisite for change of behaviour, an increase in knowledge about HIV does not predict behavioural change [10,11]. Recent studies on HIV among young adults in Ghana has predominantly focused on public universities [5] with paucity of data on private university students, although there are about 30 private universities in Accra alone as indicated by the National Accreditation Board - a regulatory agency of the Ministry of Education that facilitates the establishment of both public and private tertiary institutions [12].

According to the Ghana AIDS Commission [13] there has been nearly universal awareness of HIV since 2003, but comprehensive knowledge has been lagging behind. The same report indicated that only 28.3% of females aged 15–24 and 34.2% of males were able correctly identify factual information and reject common misconceptions [13]. This reflected a slight increase of 3.2% and 1.2% for females and males respectively from the previous year. This statistics has health implications for the formulation of policy and HIV prevention interventions.

Access to HIV testing is considered as a cornerstone to the strategic framework adopted by Ghana for HIV control. As a result the Government of Ghana has since introduced and implemented various programmes to increase testing. Notwithstanding these interventions, HIV testing uptake is still low and unknown to many Ghanaians [14,15], due to the fear of the testing outcome and HIV related stigma [16]. There is very little information on HIV testing among university students and the factors that influence them to test for HIV. It is also not clear if the factors that influence the general population to test for HIV [17,18] can be attributed to HIV testing uptake among private university students. Despite young adult’s vulnerability to HIV infection due to risky sexual behaviours, very few studies have examined HIV/AIDS knowledge and the uptake of HIV testing among private university students in Accra, although HIV Counselling and Testing (VCT) and knowledge about HIV are some key strategies in the prevention and control of HIV/AIDS in Ghana.

The main objective was to determine the level of HIV/AIDS knowledge and to explore factors associated with the use HIV counselling and testing among private university students in Accra, Ghana. The outcome of the findings could help in the formulation of policy on access of HIV counselling and testing services to university students in Ghana, as such age-groups are at greater risk for HIV infection.

## Method

### Sample

Three hundred and twenty-four (324) undergraduate university students (43.8% females and 56.2% males) from a private tertiary institution in Accra, Ghana, with an age range of 17 to 37 were conveniently sampled and included in the study. Convenience sampling was used due to easy access to students and cost effectiveness. The students were enrolled in 10 different undergraduate academic programmes in the participating university. The private university has four main satellite campuses with over 2000 students. The university offers predominantly undergraduate with few postgraduate programmes in three faculties namely Informatics and Engineering Sciences, Business and Economics, and Social Sciences. The sample size represents 94% of response rate from eligible prospective individuals approached to take part in the study.

### Measures

A self-administered AIDS Knowledge and Attitude Inventory [19] validated within the Ghanaian context (5, 16) was used to measure participants knowledge about HIV/AIDS. Questions were asked to assess respondents' knowledge about the HIV and AIDS. There were three sets of questions assessing AIDS knowledge in different formats. The first set consisted items assessing knowledge of definition and causation of HIV/AIDS. The second set contained questions assessing modes of AIDS virus transmission, and the third set of questions regarding AIDS symptoms and preventive measures. The summation of these scores formed the basis for data analysis. The possible score ranges from 0 to 12. Higher scores (7–12) indicate more accurate knowledge on HIV/AIDS. The respondents' knowledge of HIV/AIDS was assessed by assigning a score of 1 to each correct answer of 12 yes/no HIV/AIDS related questions. The instrument had questions on demographic characteristics to measure age, level, gender sex, religion, and marital status.

Three main questions were asked that to assess the knowledge and usage of HIV Voluntary Counselling and Testing (VCT). These include questions on whether they know where to access VCT services (no = 0, yes = 1), whether they have ever tested for HIV (no = 0, yes = 1) and if they would test for HIV in the future (no = 0, yes = 1).

Participants were asked to indicate whether they had received AIDS information in the past 6 months from any of 5 sources (e.g. internet, radio, television, magazines/newspapers and parents). Students were also asked to check sources that could provide the most reliable information on AIDS.

### Data collection

After permission was granted from the registrar, university lecturers were approached to allow the researcher to use the last 20 minutes of their lecture time. Out of 20 lecturers contacted, only two declined because they were not prepared to offer the requested time of their lecture period to be used for the study. Having explained the nature of the research, the researcher then provided explanation of the study as well as the purpose of the study. The students were informed that the survey was anonymous and were assured of the confidentiality of their responses. The questionnaire was completed in the presence of the researcher during the last 20 minutes of a 2-hour lecture. Over two-thirds (83%) of the students were in their first and second years because most of the senior lecturers that declined were handling the third and final year students. Participation was voluntary and students had the opportunity to refuse participation. Five (5) questionnaires were left on the desk without being filled whilst 14 students did not answer any item at all on the questionnaire. Thus, non response was 19 (6%).

Ethical permission was obtained from Regent University College of Science & Technology ethical review board. Informed consent from students was a requirement for participation with emphasis on voluntary participation, confidentiality and anonymity of the information provided.

### Statistical analysis

An independent sample *t*-test and Analysis of variance (ANOVA) was used to compare the differences in mean of the knowledge on HIV/AIDS according to the various demographic characteristics. In addition, Chi-square (***χ***^**2**^) was used to explore the factors associated with having taken an HIV test and the desire to be tested in the future as well as the sources of HIV/AIDS information. To assess the relative contribution of each of these predictor variables, a logistic regression analysis was carried out. Variables with a p < 0.05 on bivariate analyses were included in the logistic model and categorical variables were re-coded into a binary form. These variables were entered into the model using a block entry approach. Odds ratios (OR) and 95% confidence intervals (CI) were produced for each predictor variable. P-values <0.05 were considered statistically significant. The Statistical Package for the Social Sciences (SPSS) version 19.0 was used for data analysis.

## Results

### Sample characteristics

Table 1 shows the characteristics of the sample in the study. The sample consisted of 43.8% females and 56.2% males within the ages of 17–37 (*M* = 22.91, *SD* = 3.79). Majority of the participants (85.6%) were not married, (96%) were Christians and over half of them (52%) were first year students.

**Table 1:** Socio-demographic information of the participants (n = 324)

**Variables**	**N**	**%**
*Age Ranges*		
17 – 20	93	28.7
21 – 25	170	52.5
26 – 30	47	14.5
31+	14	4.3
*Gender*		
Male	182	56.2
Female	142	43.8
*Marital Status*		
Single	196	60.5
Married	32	9.9
In relationship	96	29.6
*Religious Affiliation*		
Christian	311	96.0
Muslims	13	4.0
*Years in School*		
First year	168	52.0
Second year	102	31.0
Third year	54	17.0

### HIV transmission and prevention knowledge

Fifty-six percent of the participants identified unprotected sexual intercourse with infected persons as a means of transmission, 44% identified sharing needles/syringes with infected persons and 25% identified mother- to child transmission. Knowledge of the modes of HIV transmission was high as majority of the respondents (96%) were able to correctly identify one or more modes of HIV of transmission. Half (162, 50%) were able to identify more than two routes of transmission, 78 (24%) could identify three or more routes and 72 (22%) could identify only a single way of HIV transmission. Only 4% of the participants in the study were unable to identify any routes of transmission.

Knowledge of HIV prevention appeared moderately high as respondents knew that condom usage (78%), abstinence from casual sex, (70%), avoiding sharing of sharp objects (63%) and being faithful to a partner (57%) were some of the ways of preventing HIV infection. Knowledge of treatment for HIV/AIDS was equally high as 89% of the respondents indicated that they know that there was no cure for AIDS, and 82% indicated that one cannot always say by merely looking if someone is infected with the virus.

### General HIV knowledge across the various demographic characteristics

The analysis of data revealed that students had a good to excellent knowledge about HIV/AIDS. The total HIV knowledge score ranged from 3 to 12 (M = 7.77; SD = 2.21). When the data was grouped according to high score (7–12) and low scores (0–6), approximately 91 (28%) had low knowledge scores whilst majority of the students had high knowledge (233, 72%). For the test of significance, an independent sample *t*-test and Analysis of variance (ANOVA) were used to compare the differences in mean of the knowledge on HIV/AIDS according to the various demographic characteristics as shown in Table 2. The results of an independent sample *t*-test which compared the differences in the mean knowledge on HIV/AIDS according to gender and religious affiliation indicated that female students (M = 8.11, SD = 2.17) had more knowledge about HIV/AIDS than their males counterpart (M = 7.5, SD = 2.21) [t (322) = 2.40, p = 0.017]. Although Muslim students scored higher on HIV/AIDS knowledge (M = 8.54, SD = 1.45), than Christian students (M = 7.74, SD = 2.23), this difference was not strong enough to yield statistical significance [t (322) = 2.27, p = 0. 0.203].

**Table 2:** Differences in HIV/AIDS knowledge by demographic characteristics

**Variables**	**Mean (SD)**	**Test values**	***p*-value**
*Age Ranges*		6.26^a^	0.0001
17 – 20	7.02 (2.04)		
21 – 25	8.06 (2.25)		
26 – 30	8.38 (2.26)		
31+	7.29 (0.99)		
*Gender*		2.40^b^	0.017
Male	7.52 (2.21)		
Female	8.11 (2.17)		
*Marital Status*		4.86^a^	0.008
Single	7.47 (2.25)		
Married	8.22 (1.43)		
In relationship	8.25 (2.43)		
*Religious Affiliation*		2.27^b^	0.203
Christian	7.74 (2.23)		
Muslims	8.54 (1.45)		
*Years in School*		1.37^a^	0.257
First year	7.80 (2.22)		
Second year	7.96 (2.16)		
Third year	7.36 (2.27)		

^a^ F-values from one-way ANOVA.

^b^ Independent *t*-test values.

The One-Way Analysis of variance (ANOVA) indicated that there was a statistically significant difference in the HIV/AIDS Knowledge scores for the four age groups [F (3, 321) = 6.26, p = 0. 0001]. Post-hoc test comparisons using the Turkey HSD test indicated that the mean score of the 16 – 20 years group (M = 7.02, SD = 2.04) was significantly different from both the 21 – 25 years group (M = 8.06, SD = 2.25) and the 26 – 30 years group (M = 8.38, SD = 2.26). The 31+ year group did not differ significantly from with any of the age groups.

The ANOVA results also indicated a statistically significant difference in the HIV/AIDS knowledge scores for the marital status of the participants [F (3, 321) = 4.86, p = 0. 008]. Post-hoc test comparisons using the Turkey HSD test indicated that the mean scores for student who were single (M = 7.47, SD = 2.25) was significantly different from those who were “in relationships” (M = 8.25, SD = 2.43). Students who were married (M = 8.22, SD = 1.43) did not differ significantly from either single or students in relationship.

### Sources of HIV/AIDS information

**Figure 1:**
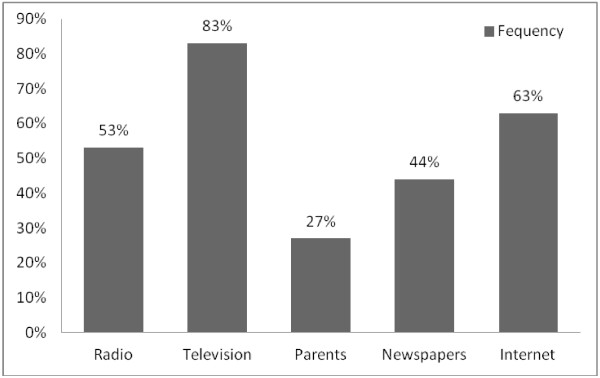
The frequency of the sources of HIV/AIDS information in the past 6 months by participants.

As shown in Figure 1, approximately 83% of the sample reported having received information about HIV/AIDS from the television, 63% from the internet, 53% from radio, whiles newspapers/magazines and parents accounted for 44% and 27%, respectively. More female students (47%) were more likely than males (41%) to rely on the newspapers/magazines for information. A participant who received any information about HIV transmission from the radio or newspapers was more likely to identify more routes of HIV transmission [χ2 (3, N = 324) = 29.75, *p* = .000] and [χ2 (3, N = 324) = 13.91, *p* = .003] respectively. Other sources that predicted HIV transmission knowledge included parents [χ2 (3, N = 324) = 12.26, *p* = .007] and television [χ2 (3, N = 324) = 11.86, *p* = .008].

### Counseling and testing for HIV

Over 95% of the students were knowledgeable about where to get an HIV test, but only 45.4% had tested for HIV. Over half (54.6%) of the participants had not tested for HIV prior to the study. Additionally, 62.7% of the participants indicated they would test for HIV in the future, with more males (67%) showing more willingness than females (33%). The data in Table 3 indicates that the variable “age” was significantly associated with having tested for HIV (*p* = 0.004). The distribution by age further indicated that those who have had an HIV test was highest among students aged 21–25 (52%) and reduces to 18% among 26–30 year olds and to only 8% among the 31+ year group. HIV testing among the sample therefore declines with age: the older the student, the less likelihood of testing for HIV. The sex of the student was also significantly related to the willingness to have HIV test in the future (*p* < 0.001). Males (67%) were more likely to have an HIV test in the future than females (33%).

**Table 3:** Demographic characteristics and their association with HIV test and intention to test for HIV

**Characteristics**	**Have ever tested for HIV (N = 324)**	**Will test for HIV in the future (N = 324)**				
	**Yes**	**No**	**χ2**	**Yes**	**No**	**χ2**
	**N (%)**	**N (%)**	**( *p- *value)**	**N (%)**	**N (%)**	**( *p- *value)**
*Age Ranges*			**0.004**			0.115
17– 20	32 (21.8)	61 (34.5)		55 (27.1)	38 (31.4)	
21 – 25	77 (52.4)	93 (52.5)		111 (54.7)	59 (48.8)	
26 – 30	27 (18.3)	20 (11.3)		32 (15.8)	15 (12.4)	
31+	11 (7.5)	3 (1.7)		5 (2.4)	9 (7.4)	
*Gender*			0.748			**0.001**
Male	84 (57.1)	98 (55.4)		136 (67.0)	46 (38.0)	
Female	63 (42.9)	79 (44.6)		67 (33.0)	75 (62.0)	
*Marital Status*			**0.001**			**0.046**
Single	87 (59.2)	109 (61.6)		133 (65.5)	63 (52.1)	
Married	24 (16.3)	8 (4.5)		16 (7.9)	16 (13.2)	
In relationship	36 (24.5)	60 (33.9)		54 (26.6)	42 (34.7)	
*Religious Affiliation*			**0.027**			0.209
Christian	145 (98.6)	166 (93.8)		197 (97.0)	114 (94.2)	
Muslims	2 (1.4)	11 (6.2)		6 (3.0)	7 (5.8)	
*Years in School*			0.416			0.197
First year	79 (53.7)	89 (50.3)		113 (55.7)	55 (45.5)	
Second year	41 (27.9)	61 (34.5)		58 (28.6)	44 (36.4)	
Third year	27 (18.4)	27 (15.3)		32 (15.7)	22 (18.1)	
*Knowledge of a place to get tested*			**0.003**			0.633
Yes	141(95.9)	153 (86.4)		183 (90.1)	111 (91.7)	
No	6 (4.1)	24 (13.6)		20 (9.9)	10 (8.3)	
*Routes of HIV transmission Identified*			**0.001**			0.762
None	-	12(6.8)		6(3.0)	6(5.0)	
Single route	24(16.3)	48(27.1)		47(23.2)	25(20.7)	
Two routes	84(57.1)	78(44.1)		100(49.3)	62(51.2)	
Three or more routes	39(26.5)	39(22.0)		50(23.5)	28(23.1)	
*HIV knowledge*			0.570			0.203
Low (0–6)	39(26.5)	52(29.4)		62(30.5)	29(24)	
High (7–12)	108(73.5)	125(70.6)		141(69.5)	92(76)	

The results in Table 3 further indicate that marital status was significantly associated with having taken an HIV test (*p* < 0.001) and willing to test for HIV in the future (*p* = 0.04). A large proportion of the students who were single (59%) were more likely to have taken an HIV test than those “in-relationship” (25%) and married (16%). Additionally, a greater proportion of never married (single) students (66%) would like to test for HIV in the future as compared to those “in relationship” (27%) or married (7%) students. There was also an association between knowing where to get tested and taking an HIV test (*p* = 0.003). Additionally, religion was also associated with having taken an HIV test (*p* = 0.027).

Table 4 shows binary logistic regression of predictors for ever taken an HIV test and willingness to test for HIV in the future. Respondents who were single are six times (OR = 5.87; 95% CI = 1.76 – 19.56, p = 0.004) more likely to have ever taken an HIV test than those married/in relationship. Additionally, students who identified two routes of HIV transmission (OR = 2.0; 95% CI = 1.03 – 3.87, p = 0.040) were two times more likely to have ever taken an HIV test than those who identified a single or three routes of HIV transmission.

**Table 4:** Logistic regression of the predictors of ever tested for HIV and intention to test for HIV in the future

**Variables**	**Coefficients (β)**	**SE**	**Odds Ratio (OR)**	**95% CI**	***p*-value**
**Have ever tested for HIV**					
Age (17–20 years vs. others)	0.521	0.27	1.68	1.01 – 2.850	0.050
Marital Status (single vs. others)	1.770	0.61	5.87	1.76 – 19.56	0.004
Religion	−1.596	0.80	0.20	0.04 – 0.96	0.045
Knowledge of a place to get tested	2.00	0.58	7.39	2.38 – 22.98	0.001
Routes of HIV Transmission	−0. 69	0.34	2.00	1.03 – 3.87	0.040
**Willingness to test for HIV in the future**					
Gender	1.12	0.25	3.20	1.96 – 5.24	< 0.000
Marital Status (single vs. others)	0.13	0.28	1.14	0.66 – 1.96	0.636

Dependent variables: yes = 1, no = 0.

Independent variables: age (17–20 years = 1, others = 0) marital status (single = 1, others = 0), sex (male = 1, female = 2), religion (Christian =1, Muslim = 0), knowledge of a place to get tested (yes = 1, no = 0), routes of HIV transmission (two routes of transmission = 1, others = 0).

The results in Table 4 further indicates that knowing a place where HIV can be tested (OR = 7.39; 95% CI = 2.38 – 22.98, p = 0.001) and being a student aged 17–20 years (OR = 1.68; 95% CI = 1.01 – 2.85, p = 0.050) were respectively associated with the odds of having had an HIV test. Males were three times more likely to be willing to take an HIV test in the future than females (OR = 3.20; 95% CI = 1.96 – 5.24, p < 0.000).

## Discussion

Among the study participants, results indicated that knowledge scores about HIV, including the cause, mode of transmission, and prevention of the disease, were high, with an average score of 7.70 out of a total of 12.0. Over 90% of the students have heard about HIV/AIDS. The result also revealed a significant gender differences in HIV knowledge among university students in Ghana, with females being more knowledgeable than males, but males were more likely to have to take an HIV test in the future than females. Participants who were single were more likely to have ever taken an HIV test than those married / in relationship. Knowing a place where to be tested and being a Christian increases the likelihood to have ever had an HIV test. Majority of the students reported having received AIDS information from both print and electronic media, but few of them received such information from parents.

The high level of HIV knowledge among the students as revealed in this study could be attributed to sustained and improved health education programmes. Over the past decade the government of Ghana through the Ghana AIDS Commission and its development partners increased public awareness on the causes and preventive of STIs including HIV/AIDS. Such government effort should be complemented by the establishment of more HIV/AIDS Clubs in tertiary institutions. This should be scaled up to cover all secondary schools as well. The main aim of HIV/AIDS Clubs is to train young tertiary students to effectively contribute to HIV/AIDS awareness on their respective campuses as peer educators. This has the potential of helping more students to become knowledgeable about prevention, thereby facilitating positive behaviour change.

The level of HIV knowledge differed between males and females in this study. This finding is consistent with studies conducted in Nigeria [20,21] which found that AIDS knowledge differs on the basis of gender among university students. However, [22] had indicated no gender differences on HIV knowledge and attitudes among university students in Nigeria. This gender difference found in this study could be explained that most female students in private universities are generally willing to discuss and respond to questions and issues related to sexuality than their male counterparts. Additionally, female university students may be more concerned about their health more than males, thus seeking to know more about issues related to health including HIV/AIDS.

The results suggest that majority of student’s access HIV/AIDS information in one way or the other. Public media: both print and electronic (e.g. television, internet, radio) seem to be the main source of information about HIV/AIDS to participants in the study. Despite the fact that a large part of the sample probably spends most of their time in their community (with immediate family and friends) little information has been gained from parents. Consistent with previous studies in Ghana [23] and Nigeria [21,24], private university students in Ghana appear to rely on both print and electronic media as the major sources of HIV/AIDS information. On the evidence of the high score on HIV/AIDS knowledge scores of the study participants, it can be assumed that the information they receive through the media is understood.

Mass-media campaigns utilizing television radio, posters and billboards have been shown to be more effective for addressing specific issues [25]. They have also been proven to be effective in increasing knowledge, improving self-efficacy to use condoms, influencing social norms, increasing the amount of interpersonal communication and raising awareness of health services [25]. These media therefore have an important role to play in raising AIDS awareness among young adults including university students. The use television and radio for the dissemination of HIV/AIDS information should be intensified since most of the youth as indicated in this study used that medium. Additionally, with the rapid usage of the internet by adolescents and young adults, the Ghana AIDS Commission should encourage its development partners (Non-Governmental Organizations, Community Based Organizations, etc.) to provide up to date health related HIV information on their websites. Encouraging such organizations to use social media networks would have the potential of attracting the youth to their various websites for vital information concerning HIV/AIDS and other health reproductive issues.

Parents were not a major source of HIV information as only 27% of the participants indicated they received such information from them. Discussion of sexual issues between parents and their children are rare in Ghana, due to the fact that the Ghanaian culture has more conservative, religious and traditional beliefs on issues of sexuality, condom usage and marriage [26]. It is also possible that the older generation had not received any information on sex education, making it difficult for them to approach the issue as parents themselves. Furthermore, residential pattern and family structure might reduce the opportunity to discuss sensitive topics like sex. Such finding highlight the need for parents to should discuss reproductive sexual issues with their children and young adults, because a strong adult protective shield for young people has been shown to decrease their risk of HIV infection [27].

Another interesting finding of the study is that over 90% of the students were knowledgeable about where to get an HIV test, but only 45% had tested for HIV. This finding is consistent with a previous study [5] which indicated that majority of public university students had not taken an HIV test. The unwillingness of students to take HIV test could be attributed to fear, anxiety and stigma as well as discrimination associated with the counseling and testing and AIDS respectively. Fear of stigma have been shown to influence young adults to become less likely to engage in preventive behaviours [2] and an increase in knowledge about HIV does not predict behavioural change [10,11]. The fact that over 90% of the participants were not married (single and “in relationship”) raises a lot of health concerns as only 39% and 56% of females and male students respectively indicated they would offer themselves for a test in the future. Although knowing where to test for HIV significantly increased the likely of getting tested, it does not necessarily influence their desire to get tested in the future. The unavailability of HIV counseling and testing facilities on various public and private university campuses in Ghana could be a possible for factor for the current behavioural trend.

The study revealed that the age and marital status of students are crucial in determining whether a student had tested for HIV and willing to test for HIV in the future. Students who were not married (single or in-relationship) (84%) had tested for HIV and 93% were willing to test for HIV in the future than those who were married. Additionally, HIV testing among the sample has been shown to reduce with age: the older the student, the less likelihood of testing for HIV. This result is encouraging but efforts to stimulate HIV testing among students in tertiary institutions in Ghana should target the fresh students, and they should be encouraged to undertake periodic testing for HIV. The few numbers of married students who have tested for HIV and willing to test in the future would not be problematic so far as they remain faithful in their marriages.

The relative impact of religion on HIV counseling and testing has been reiterated by previous researchers in Ghana [18,28,29]. Some religious groups in Ghana for instance make HIV testing a pre-requisite for the celebration of marriage ceremonies, and therefore encouraging members to get tested for HIV. This could explain why more Christians were willing to be tested for HIV in this study. Although this practice was criticized by some organizations when religious bodies started such practices, it is now believed that VCT services can facilitate behaviour change, as those who are HIV negative before marriage may subsequently resort to condom use in order to remain so[30]. These therefore give credence to the involvement of religious leaders and institutions in the prevention of HIV/AIDS programmes in Ghana.

### Limitations of the study

Even though every effort was made to maintain the quality of the data, the study has shortfalls that should be noted when interpreting the results. One of the limitations of this study was cross – sectional data; therefore, causal interpretation of the results cannot be established. Furthermore, it was conducted among only one private university college thus the results cannot be generalized to the entire population of undergraduate students in Ghana due to the relative sample size and the non-probability sampling technique used. The limitation of self-reported data also apply to the current findings, as accuracy of the data cannot be certain, as a result of the social desirable nature of the subject under study. Despite these shortcomings, this study fills a gap in research in the area of HIV/AIDS knowledge among private university students, and the influence of demographic variables associated with HIV counseling and testing.

Future studies should investigate student’s risky sexual behaviour pattern and HIV risk perfection to help provide a comprehensive understanding of sexual issues among students in institutions of higher education in Ghana. A qualitative approach could be adopted to explore the possible reasons for not willing to test for HIV, especially female students.

## Conclusion

Based on the findings of the study, the relatively small number of students willing to test for HIV in the future, should be of great concern to public health practitioners. University administrators and authorities can actively help to develop and implement HIV education and prevention strategies in their campuses. HIV intervention programmes must not only provide accurate information, but must be made to help equip private university students, especially females to test for HIV consistently.

## Competing interest

The author declares that there are no competing interests.

## Authors’ contribution

KOA designed the study, analysed the data and wrote the manuscript.
